# Associations between non‐daily smoking and motivation to stop smoking: A population study in England 2021–2024

**DOI:** 10.1111/add.70159

**Published:** 2025-08-07

**Authors:** Sarah E. Jackson, Jamie Brown, Lion Shahab, Sharon Cox

**Affiliations:** ^1^ Department of Behavioural Science and Health University College London London UK; ^2^ Behavioural Research UK Edinburgh UK

**Keywords:** age, motivation, non‐daily smoking, population study, smoking cessation, socioeconomic position

## Abstract

**Background and aims:**

An increasing proportion of adults in England who smoke cigarettes do not smoke every day and may see quitting smoking as less important than those who smoke daily. This study aimed to examine whether motivation to stop smoking differs between those who smoke cigarettes daily vs. non‐daily, and to explore differences in this association by relevant sociodemographic, smoking and vaping‐related factors.

**Design and setting:**

Observational study using data drawn from the Smoking Toolkit Study, a representative cross‐sectional survey in England, 2021–2024.

**Participants:**

13 277 cigarette smokers (≥16 y).

**Measures:**

Outcome variables were level of motivation to stop smoking (Motivation to Stop Scale), analysed as a 7‐level ordinal variable and dichotomised to assess (1) no desire to stop smoking and (2) high motivation to stop smoking. The exposure variable was daily vs. non‐daily smoking. Covariates and potential moderators were age, gender, socioeconomic position, presence of children in the household, strength of urges to smoke, vaping status, harm perceptions of e‐cigarettes vs. cigarettes and survey year.

**Results:**

Non‐daily (vs. daily) smoking was associated with greater motivation to stop smoking [adjusted odds ratio (OR_adj_) = 1.22 (95% confidence interval, CI = 1.13–1.32)]. Non‐daily smokers were both less likely than daily smokers to report no desire to stop smoking [40.4% vs. 44.0%; OR_adj_ = 0.85 (95% CI = 0.77–0.95)] and more likely to report high motivation to stop smoking [21.0% vs. 14.8%; OR_adj_ = 1.78 (95% CI = 1.55–2.03)]. These differences in motivation—especially in the odds of reporting no desire to stop smoking—between non‐daily and daily smokers were more pronounced among those who were older and less advantaged. Differences were less pronounced among those who reported no urges to smoke, those who vaped and those who perceived e‐cigarettes to be less harmful than cigarettes.

**Conclusions:**

In England, adults who smoke cigarettes non‐daily appear to tend to be more motivated to quit smoking than those who smoke every day, especially among older and less advantaged people.

## INTRODUCTION

The government has set an ambitious target for England to become ‘smokefree’ by 2030, defined as an adult smoking prevalence of 5% or less [1, 2]. Achieving this will depend on large numbers of those who currently smoke making a successful quit attempt. According to the Annual Population Survey (APS), which the government uses as its official estimate of smoking prevalence, 11.6% of adults in England smoked cigarettes in 2023 [3]. However, the way smoking status is assessed in the APS is likely to miss some non‐daily smokers [4]. The Smoking Toolkit Study (STS), another nationally representative survey that asks explicitly about daily and non‐daily smoking, estimated a higher smoking prevalence of 14.6% in 2023 [4]. Since 2020, this divergence between APS and STS estimates has grown, reflecting a growing trend for non‐daily smoking.

According to the STS, an increasing proportion of adults in England who smoke cigarettes do not smoke every day [5]. In a recent study [5], we found that the proportion of adult cigarette smokers in England who smoked non‐daily was relatively stable between 2006 and 2013, at approximately 10% to 11%. However, this number increased considerably over the following decade, particularly from 2020, reaching 27% by April 2024. Despite smoking non‐daily, this group still smoke approximately 21 cigarettes [i.e. just over a standard United Kingdom (UK) pack] a week, which causes important health risks [5]. The increase in non‐daily smoking was more pronounced among younger adults and among those who also vaped.

In this study, we also found that levels of motivation to stop smoking appeared to be relatively low among non‐daily smokers and declined over time (e.g. the proportion highly motivated to quit within the next 3 months decreased from 30.8% in 2006–2009 to 21.0% in 2021–2024) [5]. However, it is not clear whether this reflects a general declining trend also seen in daily smokers or whether it is specific to those who smoke non‐daily. It is important to understand whether, and if so how far, motivation to stop smoking—a key driver of quit attempts [6, 7]—differs between this relatively new population of non‐daily smokers and daily smokers in England. Any differences in motivation may need to be considered when developing public health messaging and planning for provision of cessation support.

It is plausible that motivation to stop smoking may differ between daily and non‐daily smokers. On the one hand, daily smokers typically exhibit higher levels of addiction than non‐daily smokers [8, 9] and report lower confidence in their ability to quit [10], which could suppress their motivation to stop. On the other, non‐daily smokers often underestimate the effects of smoking on their health [8, 11] and some do not even consider themselves to be smokers [10, 12], so they may be less motivated to stop smoking than those who smoke every day. The extent of any differences in motivation between daily and non‐daily smokers may vary according to other smoking and socio‐demographic characteristics, such as the strength of their urges to smoke (an indicator of their level of cigarette addiction), their age, gender, socio‐economic position and whether they have children in their household [13]. Differences in motivation between daily and non‐daily smokers may also be moderated by whether they also vape and how they perceive the relative harms of vaping compared with smoking. Non‐daily smoking is more common among those who also vape [5] and misperceptions about the relative harms of vaping compared with smoking have also increased over the last 5 years [14]. If people who vape alongside smoking think there is little or no difference in the relative harms between the products they may be less motivated to quit smoking altogether.

Using data collected within the STS from adults across Great Britain (England, Scotland and Wales), this study aimed to examine the extent to which motivation to stop smoking differs between daily and non‐daily cigarette smokers. We also explored whether this association is moderated by age, gender, socio‐economic position, presence of children in the household, strength of urges to smoke, vaping status and harm perceptions of e‐cigarettes versus cigarettes.

## METHODS

### Pre‐registration

The study protocol and analysis plan were pre‐registered on Open Science Framework (https://osf.io/rdhq7/). We made one amendment, which was to include survey year as an additional covariate in our regression analyses. In unplanned analyses, we tested the two‐way interaction between non‐daily smoking and survey year for each outcome, and analysed harm perceptions of e‐cigarettes versus cigarettes as a four‐level (rather than binary) variable to provide further insights.

### Design

Data were drawn from the STS, an ongoing monthly cross‐sectional survey of a representative sample of adults (≥16 years) in Great Britain [15, 16]. The study uses a hybrid of random probability and simple quota sampling to select a new sample of approximately 2450 adults each month. Data are collected through telephone interviews. Comparisons with other national surveys and sales data indicate the survey achieves nationally representative estimates of key variables such as socio‐demographic characteristics, smoking prevalence and cigarette consumption [15, 17].

The present analyses focused on data from participants who reported current cigarette smoking, surveyed between January 2021 and November 2024. We limited our analysis to this period to provide up‐to‐date estimates while ensuring sufficient sample sizes.

### Measures

#### Outcomes

Motivation to stop smoking was assessed with the Motivation to Stop Scale, a single‐item measure that asked: ‘Which of the following best describes you?’

1. I do not want to stop smoking
2. I think I should stop smoking but do not really want to
3. I want to stop smoking but have not thought about when
4. I really want to stop smoking but I do not know when I will
5. I want to stop smoking and hope to soon
6. I really want to stop smoking and intend to in the next 3 months
7. I really want to stop smoking and intend to in the next month

We analysed responses in three ways: (1) as an ordinal variable reflecting the level of motivation to stop smoking, and as dichotomous variables distinguishing those reporting; (2) no desire to stop smoking (responses 1–2 vs. other responses); and (3) high motivation to stop smoking (responses 6–7 vs. other responses).

In the most recent wave (November 2024), the survey included a new question that asked current cigarette smokers: ‘How much of the time in the last month have you spent thinking about the harms of smoking?’ with response options (a) I do not believe smoking is harmful; (b) not at all; (c) a little of the time; (d) some of the time; (e) a lot of the time; (f) all the time. We provided descriptive data on the proportion of daily and non‐daily cigarette smokers endorsing each response.

#### Exposure

Smoking status was assessed by asking participants which of the following best applied to them: (a) I smoke cigarettes (including hand‐rolled) every day; (b) I smoke cigarettes (including hand‐rolled), but not every day; (c) I do not smoke cigarettes at all, but I do smoke tobacco of some kind (e.g. pipe, cigar or shisha); (d) I have stopped smoking completely in the last year; (e) I stopped smoking completely more than a year ago; (f) I have never been a smoker (i.e. smoked for a year or more). Those who responded (a) were considered daily cigarette smokers and those who responded (b) non‐daily cigarette smokers. Those who responded (c–f) were excluded from the analytic sample.

#### Potential moderators

Age was analysed as a continuous variable, modelled using restricted cubic splines (see *Statistical analysis*). We also provided descriptive data by age group (16–24, 25–34, 35–44, 45–54, 55–64 and ≥65).

Gender was self‐reported and categorised as man or woman. Those who identified in another way were excluded because of low numbers (*n* < 5 within some intersections of smoking frequency and motivation to stop).

Socio‐economic position was categorised based on National Readership Survey occupational social grade classifications [18] as ABC1 (includes managerial, professional and upper supervisory occupations) and C2DE (includes manual routine, semi‐routine, lower supervisory, state pension and long‐term unemployed). Children in the household was categorised as yes (≥1) or no (0).

Level of cigarette addiction was assessed with two questions that asked participants to self‐report ratings of the strength of urges to smoke over the last 24 hours [19]. The first question asked: ‘How much of the time have you felt the urge to smoke in the past 24 hours?’ with response options: not at all, a little of the time, some of the time, a lot of the time, almost all of the time or all the time. All except those who responded ‘not at all’ were then asked: ‘In general, how strong have the urges to smoke been?’ with response options: slight, moderate, strong, very strong or extremely strong. We coded urges to smoke as 0 for those who responded ‘not at all’ to the first question and as 1, 2, 3, 4 and 5 for those who responded ‘slight’, ‘moderate’, ‘strong’, ‘very strong’ and ‘extremely strong’ to the second question. This validated measure has similar predictive value as the Fagerström Test of Cigarette Dependence and the Heaviness of Smoking Index for cessation [20].

Vaping status was assessed within three questions asking about use of a range of nicotine products:

1. ‘Do you regularly use any of the following in situations when you are not allowed to smoke?’
2. ‘Which, if any, of the following are you currently using to help you cut down the amount you smoke?’
3. ‘Can I check, are you using any of the following either to help you stop smoking, to help you cut down or for any other reason at all?’

Those who reported using an e‐cigarette in response to any of these questions were considered current vapers, else they were considered non‐vapers. While the assessment of vaping in the STS is somewhat unusual, with these questions asking about vaping in the context of smoking, we see alignment in estimates of vaping prevalence with other nationally representative surveys in England that use more straightforward language.

Relative harm perceptions of e‐cigarettes versus cigarettes were assessed with the question: ‘Compared to regular cigarettes, do you think electronic cigarettes are more, less, or equally harmful to health?’ Response options were ‘more harmful’, ‘less harmful’, ‘equally harmful’ or ‘do not know’. For our primary analyses, we dichotomised responses to less harmful versus all other responses, consistent with current evidence that e‐cigarettes are less harmful than cigarettes [21]. In an unplanned sensitivity analysis, we analysed all four response options separately. Survey year was modelled as a categorical variable from 2021 to 2024.

### Statistical analysis

Data were analysed in R v.4.4.2. We analysed data from participants who had complete data on all variables of interest. Time thinking about the harms of smoking was only included in the most recent survey wave and therefore, analysis of this variable was restricted to this wave. The STS uses raking to weight the sample to match the population in Great Britain. These profiles are determined each month by combining data from the UK Census, the Office for National Statistics mid‐year estimates and the annual National Readership Survey [15]. The following analyses used weighted data.

We estimated the proportions of daily and non‐daily smokers reporting each level of motivation to stop smoking, overall and by age, gender, socio‐economic position, children in the household, strength of urges to smoke, vaping status and harm perceptions of e‐cigarettes versus cigarettes. We also provided descriptive data on how frequently daily and non‐daily smokers reported thinking about the harms of smoking.

We used logistic regression (ordinal/binary, as relevant) to estimate the association between non‐daily smoking (vs. daily smoking) and the three motivation to stop smoking outcomes, with and without adjustment for age, gender, socio‐economic position, children in the household, strength of urges to smoke, vaping status, harm perceptions of e‐cigarettes versus cigarettes and survey year. Age was modelled using restricted cubic splines with three knots (at the 5th, 50th and 95th percentiles), to allow for non‐linear associations with motivation to stop smoking.

To test for moderation by age, gender, socio‐economic position, children in the household, strength of urges to smoke, vaping status, harm perceptions of e‐cigarettes versus cigarettes and survey year, we repeated the adjusted models with the addition of a two‐way interaction term between non‐daily smoking and each potential moderator. Each interaction was tested in a separate model. Where we found evidence of moderation (indicated by a Wald test with *P* < 0.05), we reran the adjusted model stratified by the moderator of interest to investigate the nature of the difference in association between subgroups. For stratified analyses, we categorised age as 16 to 34, 35 to 54 and ≥55 years and strength of urges to smoke as not at all, slight, moderate and strong/very strong/extremely strong to illustrate differences while reducing the number of groups for comparison.

## RESULTS

There were 14 430 cigarette smokers surveyed between January 2021 and November 2024. We excluded 202 participants who described their gender in another way and a further 951 with missing data on one or more variables (Table S1), leaving a final sample of 13 277 participants. Relative to the analysed sample, participants who were excluded were more likely to be ≥65 years of age and less likely to report moderate urges to smoke, current vaping or that e‐cigarettes were less harmful than cigarettes (Table S1).

Sample characteristics in relation to frequency of smoking are provided in Table 1. On average, non‐daily smokers were younger (28.6% vs. 12.0% age 16‐24 years) and more socio‐economically advantaged (51.5% vs. 37.9% ABC1) than daily smokers. They were also slightly more likely to be men (55.3% vs. 51.9%), but the proportion who had children in the household was similar across groups (30.2% vs. 29.0%). Non‐daily smokers reported weaker urges to smoke (36.9% vs. 6.2% reporting no urges at all in the past 24 hours) and were more likely to vape (37.2% vs. 26.0%) and to perceive e‐cigarettes to be less harmful than cigarettes (34.0% vs. 24.7%).

**TABLE 1:** Characteristics of daily and non‐daily cigarette smokers.

Characteristics	% [95% CI]	
	Non‐daily cigarette smokers (*n* = 3129)	Daily cigarette smokers (*n* = 10 148)
Age (y)		
16–24	28.6 [26.7–30.4]	12.0 [11.2–12.8]
25–34	28.7 [26.9–30.6]	23.0 [22.0–24.0]
35–44	16.9 [15.4–18.4]	18.1 [17.2–19.0]
45–54	11.0 [9.8–12.2]	17.9 [17.1–18.8]
55–64	8.7 [7.6–9.7]	14.8 [14.1–15.6]
≥65	6.1 [5.2–6.9]	14.2 [13.5–14.9]
Gender		
Men	55.3 [53.3–57.3]	51.9 [50.8–53.1]
Women	44.7 [42.7–46.7]	48.1 [46.9–49.2]
Socio‐economic position		
ABC1 (more advantaged)	51.5 [49.5–53.5]	37.9 [36.9–38.9]
C2DE (less advantaged)	48.5 [46.5–50.5]	62.1 [61.1–63.1]
Children in the household		
No	69.8 [68.0–71.7]	71.0 [70.0–72.1]
Yes	30.2 [28.3–32.0]	29.0 [27.9–30.0]
Strength of urges to smoke		
Not at all	36.9 [34.9–38.8]	6.2 [5.7–6.8]
Slight	28.4 [26.6–30.2]	19.1 [18.2–19.9]
Moderate	25.2 [23.5–27]	44.4 [43.3–45.5]
Strong	6.7 [5.7–7.7]	19.2 [18.3–20.1]
Very strong	2.0 [1.5–2.6]	6.8 [6.2–7.4]
Extremely strong	0.8 [0.5–1.2]	4.3 [3.9–4.7]
Vaping status		
Non‐vaper	62.8 [60.8–64.7]	74.0 [73.0–75.0]
Current vaper	37.2 [35.3–39.2]	26.0 [25.0–27.0]
Harm perception of e‐cigarettes vs. cigarettes		
Less harmful	34.0 [32.1–35.9]	24.7 [23.7–25.7]
Equally harmful	34.5 [32.6–36.4]	36.2 [35.1–37.3]
More harmful	18.8 [17.2–20.4]	21.4 [20.4–22.3]
Unsure	12.8 [11.5–14.1]	17.8 [16.9–18.7]

### Differences in motivation between daily and non‐daily smokers

**FIGURE 1:**
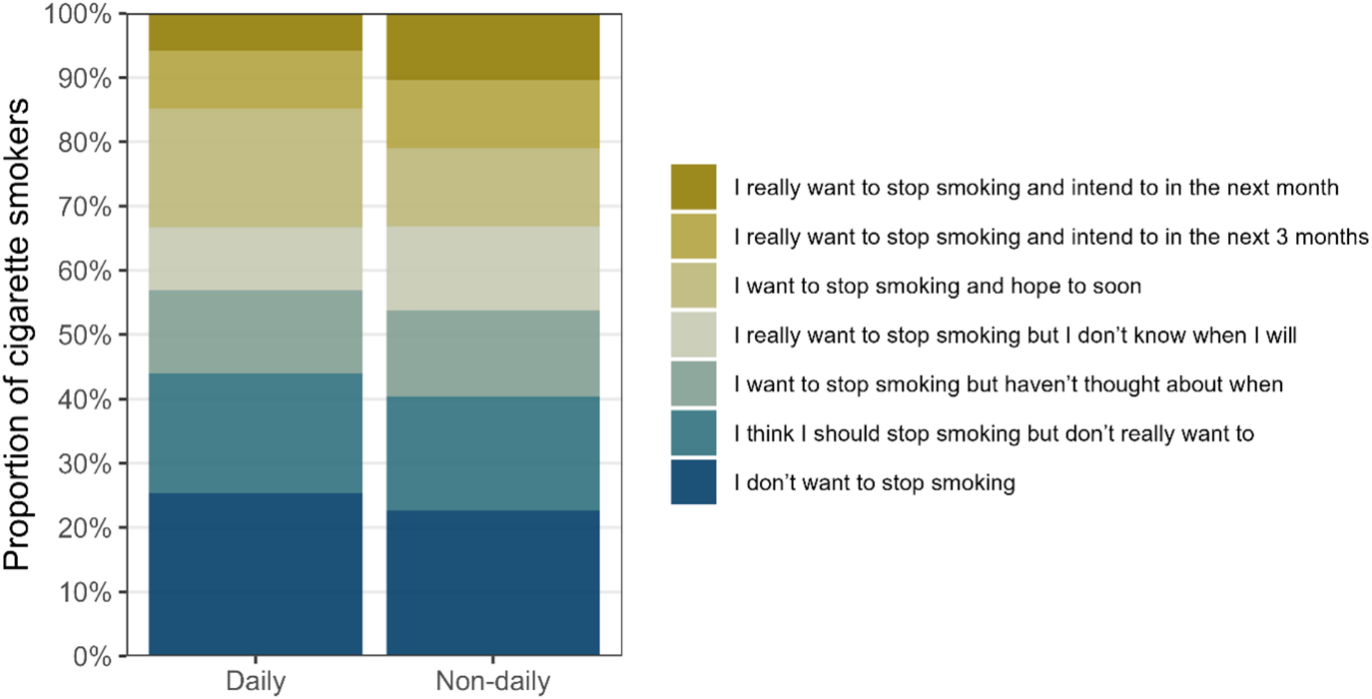
Motivation to stop smoking among daily and non‐daily cigarette smokers. Estimates with 95% CI are provided in Table 2.

Figure 1 and Table 2 summarise the proportion of daily and non‐daily cigarette smokers reporting each level of motivation to stop smoking. In unadjusted ordinal analyses, non‐daily smokers reported greater motivation to stop smoking than daily smokers [OR = 1.17 (95% CI = 1.09–1.25)]. The same pattern was observed when responses were categorised as binary variables: non‐daily smokers were less likely than daily smokers to report no desire to stop smoking [40.4% vs. 44.0%; OR = 0.86 (95% CI = 0.79–0.95)] and more likely to report high motivation to stop smoking [21.0% vs. 14.8%; OR = 1.53 (95% CI 1.36–1.71)]. These differences remained after adjusting for covariates (Table 2).

**TABLE 2:** Associations between frequency of smoking and motivation to stop.

	% [95% CI]^b^		OR [95% CI]	
	Daily cigarette smokers	Non‐daily cigarette smokers	Unadjusted	Adjusted^c^
Level of motivation to stop smoking^a^	–	–	1.17 [1.09–1.25]	1.22 [1.13–1.32]
I do not want to stop smoking	25.4 [24.4–26.3]	22.6 [20.9–24.2]	–	–
I think I should stop smoking but do not really want to	18.7 [17.8–19.5]	17.8 [16.3–19.3]	–	–
I want to stop smoking but have not thought about when	12.9 [12.1–13.6]	13.3 [12.0–14.7]	–	–
I really want to stop smoking but I do not know when I will	9.8 [9.1–10.4]	13.0 [11.7–14.4]	–	–
I want to stop smoking and hope to soon	18.5 [17.7–19.4]	12.2 [10.9–13.6]	–	–
I really want to stop smoking and intend to in the next 3 months	9.0 [8.3–9.6]	10.6 [9.4–11.8]	–	–
I really want to stop smoking and intend to in the next month	5.8 [5.3–6.4]	10.4 [9.2–11.6]	–	–
No desire to stop smoking	44.0 [42.9–45.1]	40.4 [38.5–42.4]	0.86 [0.79–0.95]	0.85 [0.77–0.95]
High motivation to stop smoking	14.8 [14.0–15.6]	21.0 [19.3–22.6]	1.53 [1.36–1.71]	1.78 [1.55–2.03]

^a^ Analysed as an ordinal variable; ORs > 1 indicate higher levels of motivation among non‐daily compared with daily cigarette smokers and ORs < 1 indicate lower levels.

^b^ Corresponding estimates for level of motivation to stop smoking stratified by age, gender, socio‐economic position, children in the household, strength of urges to smoke, vaping status and harm perceptions of e‐cigarettes vs. cigarettes are provided in Table S3 (daily cigarette smokers) and Table S4 (non‐daily cigarette smokers).

^c^ Adjusted for age, gender, socio‐economic position, children in the household, strength of urges to smoke, vaping status, harm perceptions of e‐cigarettes vs. cigarettes (less harmful vs. other perception) and survey year.

Of those who provided data (*n* = 173), 69.8% of non‐daily smokers reported thinking about the harms of smoking in the past month (29.8% a little of the time, 26.2% some of the time, 9.1% a lot of the time and 4.5% all the time) compared with 64.8% of daily smokers (18.2%, 23.7%, 16.8% and 6.1%, respectively) (Table S2).

### Moderation by socio‐demographic, smoking‐ and vaping‐related factors

**FIGURE 2:**
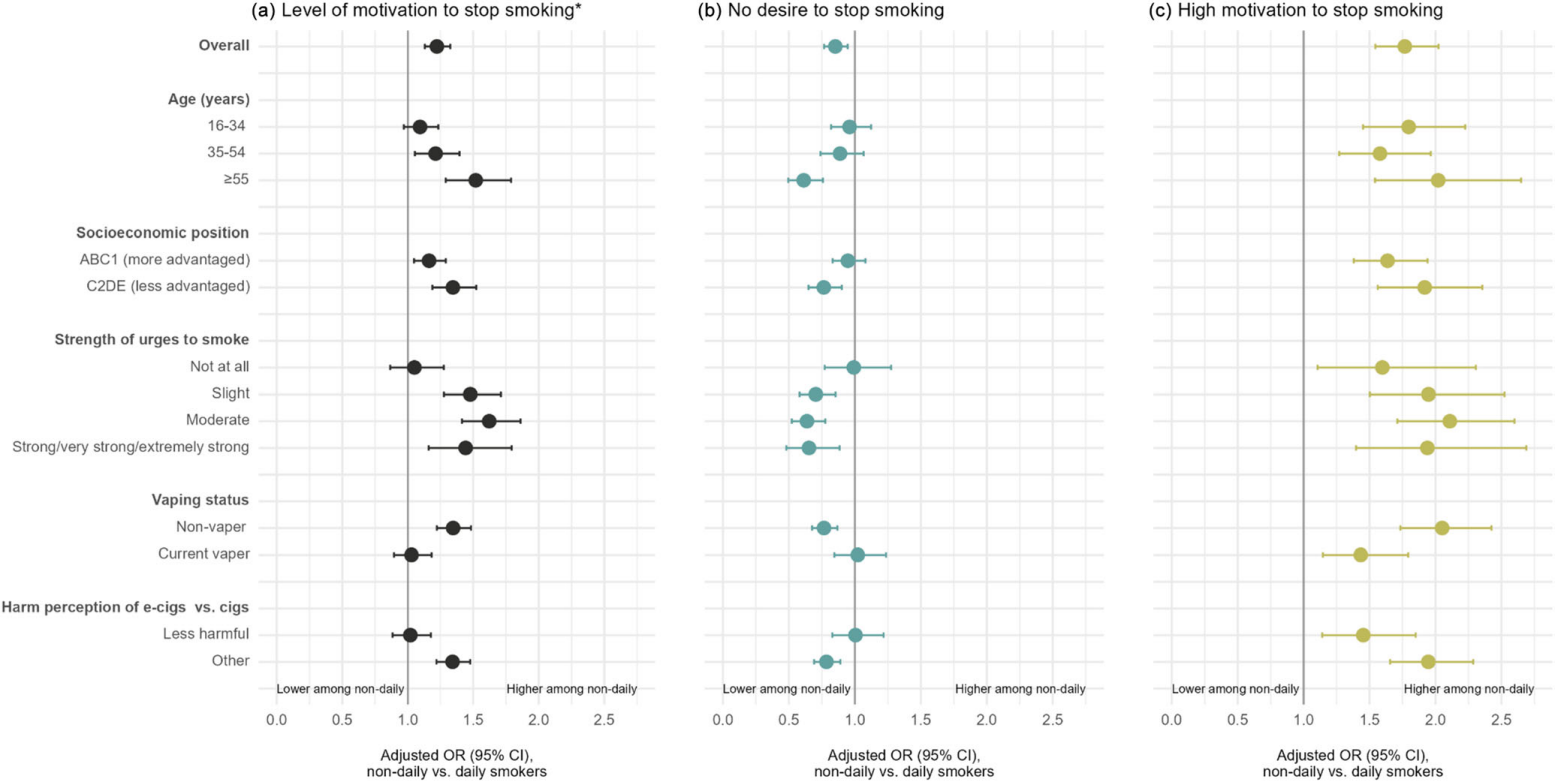
Associations between frequency of smoking and motivation to stop smoking, overall and by moderating variables. *Analysed as an ordinal variable. Overall estimates are reported in Table 2 and estimates within subgroups in Table S7.

Estimates of motivation to stop smoking stratified by age, gender, socio‐economic position, children in the household, strength of urges to smoke, vaping status and harm perceptions of e‐cigarettes versus cigarettes are provided in Table S3 (daily cigarette smokers) and Table S4 (non‐daily cigarette smokers). A more detailed breakdown by harm perceptions is shown in Table S5 (daily and non‐daily cigarette smokers). There were significant interactions between non‐daily smoking and age, socio‐economic position, strength of urges to smoke, vaping status and harm perceptions of e‐cigarettes versus cigarettes (Table S6). Stratified results are presented in Figure 2 and Table S7.

Differences in motivation between daily and non‐daily cigarette smokers were greater among those who were older and less advantaged (Figure 2). Age differences appeared to be driven largely by differences in the proportion reporting no desire to stop smoking. The OR for non‐daily versus daily older smokers was 0.61 (95% CI = 0.50–0.76), indicating the odds of having no desire to stop smoking were 39% lower among non‐daily smokers. Among younger and middle‐aged smokers, the odds of having no desire to stop smoking were similar between those who smoked daily and non‐daily [OR = 0.96 (0.82–1.12) and OR = 0.89 (0.74–1.07), respectively]. Smokers from less advantaged socio‐economic positions were more likely to say they had no desire to stop smoking if they smoked daily versus non‐daily [OR = 0.76 (0.65–0.90)], but odds were similar between daily and non‐daily smokers who were more advantaged [OR = 0.95 (0.83–1.08)].

There were no notable differences in overall levels of motivation between daily and non‐daily cigarette smokers among those who reported experiencing no urges to smoke, those who currently vaped or those who perceived e‐cigarettes to be less harmful than cigarettes (Figure 2). This was driven by the odds of having no desire to stop smoking being similar between daily and non‐daily smokers in these subgroups. Non‐daily smoking was associated with higher odds of high motivation to stop smoking across all subgroups. Differences between daily and non‐daily smokers were observed for all motivation outcomes among those reporting urges to smoke (with no clear differences in the size of the association according to the strength of these urges), those who did not vape and those who did not perceive e‐cigarettes to be less harmful than cigarettes (with a broadly similar pattern of results across those who believed they were equally or more harmful than cigarettes or who were unsure of the relative harms) (Table S7).

Associations between non‐daily smoking and motivation did not differ significantly by survey year.

## DISCUSSION

In this representative sample of cigarette smokers in England, those who smoked non‐daily reported greater motivation to stop smoking than those who smoked every day. Non‐daily smokers were both less likely than daily smokers to report no desire to stop smoking and more likely to report high motivation to stop smoking. Differences in motivation—especially in the odds of reporting no desire to stop smoking—between non‐daily and daily smokers were more pronounced among those who were older and less advantaged. Differences were less pronounced among those who reported no urges to smoke, those who vaped and those who perceived e‐cigarettes to be less harmful than cigarettes.

We had thought it plausible that non‐daily smokers may see quitting as less urgent than daily smokers because evidence suggests they may consider their smoking to be less harmful [8, 11]. However, we observed the opposite pattern: higher motivation among those who do not smoke every day. There may be several reasons for this. First, non‐daily smokers are typically less addicted to cigarettes [8, 9], which may make quitting seem more feasible and less daunting compared to daily smokers, who experience stronger withdrawal symptoms. Psychologically, non‐daily smokers may not strongly identify as ‘smokers’ [10, 12], making the prospect of quitting seem easier, and as they do not rely on cigarettes as part of their daily routines quitting may feel like a less disruptive change. Alternatively, it may be that there were a group of people who were originally daily smokers and more motivated to quit smoking and this greater motivation led them to cut down to non‐daily smoking while retaining, or increasing, their motivation to quit completely. Both longitudinal and qualitative research could help to disentangle the direction of associations.

Lower motivation to quit among daily smokers was particularly evident among those who were older and less advantaged. Older smokers were more likely than younger smokers to say that they did not want to stop smoking, and in particular, older people who smoked every day were more likely to report no desire to stop than those who smoked non‐daily. This may reflect smoking being a more deep‐rooted behaviour in this group, who likely have longer smoking histories and higher levels of addiction [22]. There may also be healthy survivor effects, whereby older people who smoke daily may not (yet) have developed smoking‐related illness, so are not highly motivated to stop. Similarly, people from less advantaged backgrounds who smoked daily were more likely to report no desire to stop smoking than those who smoked non‐daily, whereas there was little difference between daily and non‐daily smokers who were more advantaged. Quitting smoking may be less of a priority for less advantaged daily smokers because of cultural smoking norms [23] or higher levels of stress coupled with the widely held belief that smoking relieves stress [24]. Motivation to stop smoking may also be a stronger driver of behaviour change among less advantaged smokers who have fewer resources at their disposal to reduce consumption. Targeted cessation support for older and less advantaged daily smokers may be particularly beneficial in addressing these disparities.

In contrast, differences in motivation between daily and non‐daily smokers were less pronounced among those who reported no smoking urges, those who vaped and those who perceived e‐cigarettes as less harmful. This suggests that cigarette addiction and attitudes toward vaping may play a role in shaping motivation to quit, with those experiencing fewer cravings or viewing vaping as a viable alternative being less influenced by their smoking frequency. Non‐daily smoking is more common among smokers who vape [5], likely because they substitute some of their smoking with vaping—either for the purpose of cutting down to quit smoking altogether or for other reasons [25, 26]. There is high‐certainty evidence from randomised controlled trials [27] and observational studies [28, 29] that e‐cigarettes are effective for helping people to stop smoking. People who are motivated to stop smoking may, therefore, be more likely to take up vaping to support quitting, regardless of whether they smoke daily or non‐daily.

This study had several limitations. The cross‐sectional design means we cannot determine causality. While we have speculated on potential explanations, further research (e.g. longitudinal and qualitative) is needed to better understand why non‐daily smokers are more motivated to quit—and why certain subgroups of smokers are more likely to have no desire to stop smoking. The response options capturing current smoking did not specify a timeframe, and it is plausible that some of those who reported non‐daily smoking may be in the process of transitioning from daily smoking to complete cessation. Similarly, some of those who reported daily smoking may have had a recent quit attempt fail, potentially leading to low motivation. Longitudinal studies could offer useful insights into individual trajectories of daily versus non‐daily smoking and changes in motivation to quit over time. Although the data were drawn from a large, representative sample and weighted to match the population in England, there were some differences between the analysed sample and those who were excluded on the basis of missing data. In addition, as a household survey, the STS does not capture certain population groups (e.g. people experiencing homeless or living in institutions) among whom smoking patterns and motivation to quit may differ.

In conclusion, adults who smoke cigarettes non‐daily tend to be more motivated to quit smoking than those who smoke every day. With non‐daily smokers representing a growing segment of the smoking population [5] our results provide some reassurance that this subgroup of smokers are comparatively highly motivated to quit and could be targeted to achieve fast reductions in smoking prevalence if there is continued investment in tobacco control to capitalise on their motivation to quit (e.g. mass media campaigns and cessation support). However, certain subgroups of daily smokers who are less motivated to quit (e.g. those who are older and less advantaged) may benefit from targeted interventions and support to ensure progress is equitable and no group is left behind.

## AUTHOR CONTRIBUTIONS

**Sarah E. Jackson:** Conceptualization (equal); formal analysis (lead); investigation (equal); methodology (equal); visualization (lead); writing—original draft (lead); writing—review and editing (equal). **Jamie Brown:** Data curation (lead); funding acquisition (equal); investigation (equal); methodology (equal); writing—review and editing (equal). **Lion Shahab:** Funding acquisition (equal); investigation (equal); methodology (equal); writing—review and editing (equal). **Sharon Cox:** Conceptualization (equal); investigation (equal); methodology (equal); writing—review and editing (equal).

## DECLARATION OF INTERESTS

J.B. has received (most recently in 2018) unrestricted research funding from Pfizer and J&J, who manufacture smoking cessation medications. L.S. has received honoraria for talks, unrestricted research grants and travel expenses to attend meetings and workshops from manufactures of smoking cessation medications (Pfizer; J&J) and has acted as paid reviewer for grant awarding bodies and as a paid consultant for health care companies. All authors declare no financial links with tobacco companies, e‐cigarette manufacturers or their representatives.

## ETHICS APPROVAL

Ethical approval for the STS was granted originally by the UCL Ethics Committee (ID 0498/001). The data are not collected by UCL and are anonymised when received by UCL.

## PRE‐REGISTRATION

Open Science Framework (https://osf.io/rdhq7/).

## Supporting information


**Table S1.** Characteristics of included and excluded participants who reported cigarette smoking.
**Table S2.** Time spent thinking about the harms of smoking among daily and non‐daily cigarette smokers.
**Table S3.** Motivation to stop smoking within subgroups of daily cigarette smokers.
**Table S4.** Motivation to stop smoking within subgroups of non‐daily cigarette smokers.
**Table S5.** Motivation to stop smoking in relation to harm perceptions of e‐cigarettes vs. cigarettes among daily and non‐daily cigarette smokers.
**Table S6.** Moderation of associations between non‐daily smoking and motivation to stop smoking by participant characteristics.
**Table S7.** Adjusted associations between non‐daily smoking and motivation to stop smoking within population subgroups.

## Data Availability

Data are available from the corresponding author.
