# Supplementary material for: Associations between non‐daily smoking and motivation to stop smoking: A population study in England 2021–2024
Source: Addiction. 2025 Aug 7;120(12):2519–26. doi: 10.1111/add.70159 (PMC12586754; doi:10.1111/add.70159)
Supplement: Supplementary file 1 — Table S1. Characteristics of included and excluded participants who reported cigarette smoking. Table S2. Time spent thinking about the harms of smoking among daily and non‐daily cigarette smokers. Table S3. Motivation to stop smoking within subgroups of daily cigarette smokers. Table S4. Motivation to stop smoking within subgroups of non‐daily cigarette smokers. Table S5. Motivation to stop smoking in relation to harm perceptions of e‐cigarettes vs. cigarettes among daily and non‐daily cigarette smokers. Table S6. Moderation of associations between non‐daily smoking and motivation to stop smoking by participant characteristics. Table S7. Adjusted associations between non‐daily smoking and motivation to stop smoking within population subgroups. [file ADD-120-2519-s001.pdf]

Table S1. Characteristics of included and excluded participants who reported cigarette smoking

	% [95% CI]	
	**Analysed sample (complete cases)**	**Excluded (missing data on ≥1 variable)[1]**
Unweighted N	13,277	1,153
Frequency of smoking		
Daily	75.9 [75.1–76.7]	74.5 [71.6–77.4]
Non-daily	24.1 [23.3–24.9]	25.5 [22.6–28.4]
Missing, n	-	0
Age (years)		
16-24	16.0 [15.2–16.8]	17.5 [15.1–19.9]
25-34	24.4 [23.5–25.2]	19.4 [16.6–22.1]
35-44	17.8 [17.0–18.6]	16.1 [13.5–18.7]
45-54	16.3 [15.6–17.0]	16.7 [14.2–19.1]
55-64	13.4 [12.7–14.0]	12.4 [10.4–14.4]
≥65	12.2 [11.7–12.8]	17.2 [14.9–19.6]
Missing, n	-	9
Gender		
Men	52.8 [51.8–53.7]	45.0 [41.6–48.4]
Women	47.2 [46.3–48.2]	37.8 [34.5–41.1]
In another way[2]	-	17.2 [14.9–19.4]
Missing, n	-	57
Socioeconomic position		
ABC1 (more advantaged)	41.2 [40.3–42.1]	40.6 [37.5–43.7]
C2DE (less advantaged)	58.8 [57.9–59.7]	59.4 [56.3–62.5]
Missing, n	-	0
Children in the household		
No	70.7 [69.8–71.7]	73.9 [70.9–76.9]
Yes	29.3 [28.3–30.2]	26.1 [23.1–29.1]
Missing, n	-	0
Strength of urges to smoke		
Not at all	13.6 [12.9–14.3]	29.3 [24.7–33.9]
Slight	21.3 [20.5–22.1]	19.5 [15.6–23.4]
Moderate	39.8 [38.8–40.8]	23.9 [19.7–28.0]
Strong	16.2 [15.5–16.9]	10.9 [8.1–13.7]
Very strong	5.6 [5.2–6.1]	5.3 [3.2–7.3]
Extremely strong	3.5 [3.1–3.8]	11.2 [8.1–14.3]
Missing, n	-	677
Vaping status		
Non-vaper	71.3 [70.4–72.2]	78.5 [75.8–81.2]
Current vaper	28.7 [27.8–29.6]	21.5 [18.8–24.2]
Missing, n	-	0
Harm perception of e-cigarettes vs. cigarettes		
Less harmful	26.9 [26.1–27.8]	17.8 [15.3–20.3]
Equally harmful	35.8 [34.8–36.7]	24.8 [22.2–27.7]
More harmful	20.7 [19.9–21.6]	24.9 [22.1–28.0]
Unsure	16.6 [15.9–17.3]	32.5 [29.5–35.6]
Missing, n	-	0

[1] In addition to the variables shown here, we excluded *n*=283 with missing outcome data.

[2] Excluded from analysed sample due to low numbers (*n*=202).

Table S2. Time spent thinking about the harms of smoking among daily and non-daily cigarette smokers

	% [95% CI]	
**Time in the last month spent thinking about the harms of smoking[1]**	**Daily cigarette smokers**	**Non-daily cigarette smokers**
Not at all	35.2 [25.7–44.8]	30.2 [16.6–43.9]
A little of the time	18.2 [10.6–25.8]	29.8 [15.6–44.1]
Some of the time	23.7 [15.4–32.1]	26.2 [13.4–39.1]
A lot of the time	16.8 [8.9–24.7]	9.1 [0.8–17.5]
All the time	6.1 [1.8–10.3]	4.5 [0.0–11.5]

CI, confidence interval.

[1] Data are from November 2024 only (the only wave to assess this outcome) based on unweighted sample sizes of *n*=121 daily cigarette smokers, *n*=52 non-daily cigarette smokers.

Table S3. Motivation to stop smoking within subgroups of daily cigarette smokers

	Motivation to stop smoking, % [95% CI] of daily cigarette smokers						
	**I don't want to stop smoking**	**I think I should stop smoking but don't really want to**	**I want to stop smoking but haven't thought about when**	**I really want to stop smoking but I don't know when I will**	**I want to stop smoking and hope to soon**	**I really want to stop smoking and intend to in the next 3 months**	**I really want to stop smoking and intend to in the next month**
Age (years)							
16-24	21.2 [18.4–24.0]	21.2 [18.4–24.0]	17.5 [14.9–20.2]	8.4 [6.6–10.3]	20.1 [17.3–22.9]	7.1 [5.3–8.8]	4.4 [3.1–5.8]
25-34	21.8 [19.6–23.9]	18.2 [16.2–20.1]	15.5 [13.7–17.4]	12.3 [10.7–14.0]	17.4 [15.4–19.3]	9.4 [8.0–10.9]	5.4 [4.3–6.5]
35-44	23.9 [21.5–26.3]	17.2 [15.2–19.3]	13.7 [11.8–15.7]	10.7 [9.0–12.4]	18.1 [16.0–20.2]	9.9 [8.2–11.6]	6.5 [5.2–7.8]
45-54	23.7 [21.5–25.9]	16.8 [14.9–18.7]	11.0 [9.4–12.7]	10.4 [8.8–12.0]	21.2 [19.1–23.4]	9.8 [8.3–11.2]	7.1 [5.8–8.4]
55-64	26.7 [24.3–29.0]	20.8 [18.7–22.9]	9.9 [8.3–11.5]	7.2 [5.9–8.6]	18.7 [16.6–20.9]	10.4 [8.8–12.1]	6.2 [4.9–7.5]
≥65	37.4 [34.9–39.9]	19.2 [17.2–21.3]	8.8 [7.4–10.3]	7.3 [5.9–8.6]	16.1 [14.2–18.0]	6.3 [5.0–7.5]	4.9 [3.7–6.1]
Gender							
Men	27.3 [25.9–28.7]	17.7 [16.5–18.9]	13.0 [11.9–14.0]	9.6 [8.6–10.5]	18.1 [16.9–19.3]	8.4 [7.5–9.3]	6.0 [5.2–6.7]
Women	23.3 [21.9–24.6]	19.6 [18.4–20.9]	12.8 [11.7–13.9]	10.0 [9.0–10.9]	19.0 [17.7–20.3]	9.6 [8.7–10.6]	5.7 [4.9–6.4]
Socioeconomic position							
ABC1 (more advantaged)	22.5 [21.2–23.7]	20.8 [19.6–22.1]	12.5 [11.5–13.5]	10.4 [9.5–11.3]	17.8 [16.6–18.9]	10.1 [9.2–11.0]	5.9 [5.2–6.6]
C2DE (less advantaged)	27.1 [25.8–28.5]	17.3 [16.1–18.5]	13.1 [12.0–14.2]	9.4 [8.4–10.3]	19.0 [17.8–20.2]	8.3 [7.4–9.2]	5.8 [5.1–6.5]
Children in the household							
No	26.8 [25.7–28.0]	18.8 [17.8–19.8]	11.9 [11.0–12.8]	9.2 [8.5–10.0]	18.7 [17.7–19.7]	8.8 [8.1–9.5]	5.8 [5.2–6.4]
Yes	21.8 [20.0–23.7]	18.3 [16.7–20.0]	15.2 [13.6–16.8]	11.1 [9.7–12.5]	18.1 [16.5–19.8]	9.4 [8.1–10.7]	6.0 [5.0–7.0]
Strength of urges to smoke							
Not at all	41.9 [37.4–46.3]	16.4 [13.0–19.8]	12.3 [9.0–15.6]	5.4 [3.5–7.3]	14.6 [11.3–17.9]	5.2 [3.4–7.0]	4.2 [2.3–6.2]
Slight	28.1 [25.7–30.4]	17.7 [15.8–19.6]	16.2 [14.2–18.2]	9.5 [7.9–11.0]	15.5 [13.6–17.4]	8.6 [7.2–10.1]	4.5 [3.4–5.6]
Moderate	21.7 [20.3–23.1]	19.2 [17.9–20.6]	13.3 [12.1–14.4]	11.2 [10.1–12.2]	19.7 [18.3–21.1]	9.1 [8.1–10.1]	5.8 [5.1–6.6]
Strong	22.5 [20.3–24.6]	19.4 [17.4–21.4]	10.5 [8.9–12.1]	9.5 [8.0–10.9]	19.7 [17.7–21.8]	10.7 [9.1–12.3]	7.7 [6.3–9.1]
Very strong	28.1 [24.2–32.0]	19.7 [16.3–23.2]	11.7 [8.8–14.5]	7.3 [5.1–9.6]	19.2 [15.9–22.5]	8.2 [5.8–10.7]	5.7 [3.9–7.6]
Extremely strong	36.0 [30.9–41.1]	14.9 [11.2–18.7]	7.3 [4.5–10.2]	7.8 [5.1–10.4]	19.3 [14.9–23.6]	8.5 [5.5–11.4]	6.2 [3.8–8.7]
Vaping status							
Non-vaper	29.2 [28.0–30.4]	19.3 [18.2–20.3]	12.7 [11.8–13.6]	9.0 [8.3–9.8]	16.5 [15.5–17.5]	7.9 [7.2–8.6]	5.4 [4.8–6.0]
Current vaper	14.5 [12.9–16.2]	16.9 [15.2–18.6]	13.4 [11.8–15.0]	11.8 [10.3–13.2]	24.3 [22.4–26.3]	12.1 [10.6–13.6]	7.0 [5.8–8.1]
Harm perception of e-cigarettes vs. cigarettes							
Less harmful	19.2 [17.4–20.9]	20.0 [18.2–21.7]	11.4 [10.0–12.8]	11.2 [9.8–12.6]	20.7 [18.9–22.5]	10.7 [9.3–12.1]	6.9 [5.8–8.0]
Other[1]	27.4 [26.2–28.6]	18.2 [17.2–19.2]	13.4 [12.4–14.3]	9.3 [8.5–10.0]	17.8 [16.8–18.8]	8.4 [7.7–9.2]	5.5 [4.9–6.1]

CI, confidence interval. Corresponding data for non-daily smokers are provided in **Table S4**. [1] Equally harmful, more harmful, or unsure; data are provided separately for these response options in **Table S5**.

Table S4. Motivation to stop smoking within subgroups of non-daily cigarette smokers

	Motivation to stop smoking, % [95% CI] of non-daily cigarette smokers						
	**I don't want to stop smoking**	**I think I should stop smoking but don't really want to**	**I want to stop smoking but haven't thought about when**	**I really want to stop smoking but I don't know when I will**	**I want to stop smoking and hope to soon**	**I really want to stop smoking and intend to in the next 3 months**	**I really want to stop smoking and intend to in the next month**
Age (years)							
16-24	20.8 [17.7–24.0]	21.4 [18.2–24.6]	15.9 [13.1–18.7]	11.3 [8.7–13.9]	12.5 [9.9–15.2]	10.2 [7.9–12.6]	7.8 [5.7–9.8]
25-34	21.8 [18.6–25.0]	17.2 [14.3–20.0]	13.7 [11.0–16.4]	14.2 [11.5–16.9]	12.3 [9.6–14.9]	9.9 [7.5–12.3]	11.0 [8.4–13.6]
35-44	21.0 [17.0–25.0]	17.4 [13.7–21.1]	11.2 [8.1–14.2]	14.7 [11.2–18.3]	14.2 [10.8–17.5]	10.6 [7.8–13.4]	10.9 [8.0–13.8]
45-54	26.9 [21.8–32.0]	12.4 [9.0–15.9]	11.9 [8.2–15.7]	13.1 [9.3–16.9]	10.0 [6.7–13.3]	13.7 [9.8–17.6]	12.0 [8.4–15.6]
55-64	23.0 [18.0–28.0]	17.0 [12.6–21.4]	12.9 [8.4–17.4]	12.3 [8.5–16.1]	12.0 [8.1–15.9]	11.2 [7.4–15.0]	11.5 [7.5–15.6]
≥65	30.7 [24.3–37.2]	16.2 [11.1–21.3]	8.9 [4.9–12.8]	11.9 [7.1–16.7]	9.5 [5.2–13.8]	9.0 [4.9–13.0]	13.9 [9.1–18.7]
Gender							
Men	24.0 [21.7–26.3]	17.4 [15.4–19.4]	14.0 [12.1–15.9]	12.8 [11.0–14.7]	12.3 [10.5–14.1]	9.4 [7.8–11.1]	10.0 [8.3–11.7]
Women	20.8 [18.4–23.2]	18.4 [16.1–20.6]	12.5 [10.6–14.5]	13.3 [11.3–15.4]	12.1 [10.1–14.1]	12 [10.1–13.9]	10.9 [9.1–12.7]
Socioeconomic position							
ABC1 (more advantaged)	23.5 [21.5–25.5]	19.9 [18.0–21.8]	13.0 [11.3–14.6]	11.7 [10.2–13.2]	11.0 [9.5–12.5]	10.9 [9.5–12.4]	10.1 [8.6–11.6]
C2DE (less advantaged)	21.6 [19.0–24.3]	15.7 [13.3–18.1]	13.8 [11.5–16.0]	14.5 [12.2–16.8]	13.5 [11.3–15.8]	10.2 [8.2–12.2]	10.7 [8.7–12.7]
Children in the household							
No	22.6 [20.6–24.5]	18.4 [16.6–20.2]	13.6 [12.0–15.2]	13.8 [12.1–15.5]	11.1 [9.6–12.6]	10.6 [9.1–12.1]	10.0 [8.5–11.4]
Yes	22.6 [19.5–25.8]	16.5 [13.7–19.3]	12.8 [10.3–15.3]	11.3 [8.9–13.7]	14.9 [12.1–17.6]	10.5 [8.3–12.8]	11.4 [9.1–13.6]
Strength of urges to smoke							
Not at all	34.9 [31.7–38.0]	18.9 [16.3–21.5]	13.4 [11.2–15.7]	10.1 [8.0–12.2]	8.5 [6.6–10.4]	7.3 [5.5–9.0]	6.9 [5.3–8.6]
Slight	16.8 [14.0–19.5]	20.0 [17.0–22.9]	14.5 [11.8–17.2]	14.4 [11.7–17.2]	13.3 [10.7–16.0]	10.9 [8.6–13.3]	10.1 [7.8–12.4]
Moderate	13.5 [10.8–16.1]	14.8 [12.0–17.6]	12.6 [10.0–15.2]	15.9 [13.0–18.7]	15.3 [12.4–18.2]	13.5 [10.9–16.1]	14.4 [11.6–17.3]
Strong	11.4 [6.9–15.8]	17.5 [11.6–23.4]	13.3 [7.7–19.0]	12.7 [7.6–17.7]	15.8 [10.3–21.3]	17.7 [11.5–23.9]	11.6 [6.9–16.3]
Very strong	25.3 [12.0–38.6]	8.1 [1.6–14.6]	8.8 [1.6–16.1]	14.4 [3.1–25.6]	16.0 [6.1–25.8]	7.9 [0.0–16.6]	19.5 [8.0–31.1]
Extremely strong	36.4 [13.8–59.0]	14.1 [0.0–28.9]	5.7 [0.0–14.0]	10.7 [0.0–23.2]	8.2 [0.0–19.8]	6.4 [0.0–17.6]	18.5 [0.0–37.4]
Vaping status							
Non-vaper	26.2 [24.0–28.3]	17.0 [15.1–18.8]	12.6 [10.9–14.3]	12.2 [10.5–13.8]	11.2 [9.6–12.8]	9.4 [7.9–10.9]	11.5 [9.9–13.1]
Current vaper	16.6 [14.1–19.0]	19.3 [16.6–21.9]	14.6 [12.3–16.9]	14.5 [12.1–16.9]	14.0 [11.6–16.4]	12.6 [10.4–14.8]	8.5 [6.7–10.3]
Harm perception of e-cigarettes vs. cigarettes							
Less harmful	21.0 [18.2–23.8]	20.5 [17.8–23.2]	14.3 [11.9–16.8]	13.2 [10.8–15.7]	10.8 [8.7–12.9]	10.4 [8.4–12.4]	9.7 [7.5–11.8]
Other[1]	23.4 [21.3–25.5]	16.4 [14.6–18.3]	12.8 [11.2–14.5]	12.9 [11.3–14.6]	13.0 [11.2–14.7]	10.7 [9.1–12.2]	10.8 [9.3–12.3]

CI, confidence interval. Corresponding data for daily smokers are provided in **Table S3.** [1] Equally harmful, more harmful, or unsure; data are provided separately for these response options in **Table S5**.

Table S5. Motivation to stop smoking in relation to harm perceptions of e-cigarettes vs. cigarettes among daily and non-daily cigarette smokers

Harm perception of e-cigarettes vs. cigarettes	Motivation to stop smoking, % [95% CI]						
	I don't want to stop smoking	I think I should stop smoking but don't really want to	I want to stop smoking but haven't thought about when	I really want to stop smoking but I don't know when I will	I want to stop smoking and hope to soon	I really want to stop smoking and intend to in the next 3 months	I really want to stop smoking and intend to in the next month
**Daily cigarette smokers**							
Less harmful	19.2 [17.4–20.9]	20.0 [18.2–21.7]	11.4 [10.0–12.8]	11.2 [9.8–12.6]	20.7 [18.9–22.5]	10.7 [9.3–12.1]	6.9 [5.8–8.0]
Equally harmful	22.0 [20.5–23.6]	19.7 [18.2–21.2]	14.9 [13.5–16.3]	9.9 [8.8–11.0]	18.5 [17.1–20.0]	9.5 [8.5–10.7]	5.5 [4.7–6.5]
More harmful	32.4 [30.2–34.8]	16.7 [14.9–18.7]	12.9 [11.3–14.7]	8.5 [7.2–10.1]	16.4 [14.6–18.3]	7.7 [6.5–9.2]	5.3 [4.2–6.6]
Unsure	32.4 [30.0–34.9]	17.0 [15.2–19.1]	10.7 [9.2–12.5]	9.0 [7.6–10.6]	18.1 [16.2–20.3]	7.1 [5.9–8.6]	5.6 [4.5–6.9]
**Non-daily cigarette smokers**							
Less harmful	21.0 [18.2–23.8]	20.5 [17.8–23.2]	14.3 [11.9–16.8]	13.2 [10.8–15.7]	10.8 [8.7–12.9]	10.4 [8.4–12.4]	9.7 [7.5–11.8]
Equally harmful	18.9 [16.4–21.7]	16.6 [14.2–19.2]	14.5 [12.3–17.1]	13.9 [11.7–16.4]	14.6 [12.3–17.3]	10.6 [8.7–12.9]	10.9 [9.0–13.1]
More harmful	27.4 [23.4–31.8]	16.8 [13.6–20.6]	10.6 [8.0–14.0]	13.1 [10.2–16.8]	11.0 [8.5–14.1]	9.7 [7.2–12.9]	11.3 [8.7–14.7]
Unsure	29.7 [24.9–34.9]	15.6 [11.8–20.2]	11.5 [8.6–15.2]	10.1 [7.2–14.0]	11.3 [7.9–15.9]	12.2 [9.0–16.4]	9.6 [6.8–13.4]

CI, confidence interval.

Table S6. Moderation of associations between non-daily smoking and motivation to stop smoking by participant characteristics

	Level of motivation to stop smoking		No desire to stop smoking		High motivation to stop smoking	
	χ^2	*p*	χ^2	*p*	χ^2	*p*
Age	11.10	0.004	7.79	0.020	4.30	0.117
Gender	2.22	0.136	0.00	0.961	0.98	0.322
Socioeconomic position	11.27	0.001	9.25	0.002	1.34	0.247
Children in the household	0.00	0.955	0.60	0.440	0.00	0.948
Strength of urges to smoke	62.12	<0.001	34.89	<0.001	12.46	<0.001
Vaping status	15.53	<0.001	14.46	<0.001	10.28	0.001
Harm perception of e-cigarettes vs. cigarettes						
Less harmful vs. other perception	19.80	<0.001	12.04	0.001	8.12	0.004
Less harmful/equally harmful/more harmful/unsure	20.76	<0.001	12.19	0.007	9.14	0.028
Survey year	5.61	0.132	5.48	0.140	1.47	0.690

Wald test for the interaction between each characteristic and non-daily smoking in models testing associations with motivation to stop smoking, adjusted for age, gender, socioeconomic position, children in the household, strength of urges to smoke, vaping status, harm perceptions of e-cigarettes vs. cigarettes, and survey year.

Table S7. Adjusted associations between non-daily smoking and motivation to stop smoking within population subgroups

	OR_{adj} [95% CI], non-daily vs. daily cigarette smokers		
	Level of motivation to stop smoking[1]	No desire to stop smoking	High motivation to stop smoking
Age			
16-34	1.09 [0.97-1.23]	0.96 [0.82-1.12]	1.80 [1.45-2.22]
35-54	1.21 [1.05-1.39]	0.89 [0.74-1.07]	1.58 [1.27-1.96]
≥55	1.52 [1.29-1.79]	0.61 [0.50-0.76]	2.02 [1.54-2.65]
Socioeconomic position			
ABC1 (more advantaged)	1.16 [1.05-1.29]	0.95 [0.83-1.08]	1.64 [1.38-1.94]
C2DE (less advantaged)	1.34 [1.19-1.52]	0.76 [0.65-0.90]	1.92 [1.56-2.36]
Strength of urges to smoke			
Not at all	1.05 [0.87-1.27]	0.99 [0.77-1.28]	1.60 [1.11-2.31]
Slight	1.48 [1.28-1.71]	0.70 [0.58-0.85]	1.95 [1.50-2.52]
Moderate	1.62 [1.41-1.86]	0.64 [0.52-0.78]	2.11 [1.71-2.60]
Strong/very strong/extremely strong	1.44 [1.16-1.79]	0.65 [0.48-0.88]	1.94 [1.40-2.69]
Vaping status			
Non-vaper	1.35 [1.22-1.48]	0.77 [0.68-0.87]	2.05 [1.73-2.43]
Current vaper	1.03 [0.90-1.18]	1.02 [0.85-1.24]	1.43 [1.15-1.79]
Harm perception of e-cigarettes vs. cigarettes (binary)			
Less harmful	1.02 [0.88-1.18]	1.01 [0.83-1.22]	1.45 [1.14-1.85]
Other	1.34 [1.22-1.48]	0.78 [0.69-0.89]	1.95 [1.66-2.29]
Harm perception of e-cigarettes vs. cigarettes (4-level)			
Less harmful	1.02 [0.88-1.18]	1.01 [0.83-1.22]	1.45 [1.14-1.85]
Equally harmful	1.38 [1.20-1.57]	0.74 [0.62-0.88]	1.85 [1.48-2.31]
More harmful	1.33 [1.11-1.60]	0.85 [0.67-1.08]	2.07 [1.51-2.82]
Unsure	1.26 [1.02-1.55]	0.83 [0.63-1.09]	2.03 [1.43-2.89]

CI, confidence interval. OR_{adj}, odds ratio adjusted for age, gender, socioeconomic position, children in the household, strength of urges to smoke, vaping status, and harm perceptions of e-cigarettes vs. cigarettes.

[1] Analysed as an ordinal variable. ORs >1 indicate higher levels of motivation and ORs <1 indicate lower levels.

Note there were no significant interactions between non-daily smoking and either gender or children in the household (**Table S6**), so stratified results are not presented for these variables.
